# Deep characterization of females with heterozygous Duchenne muscular dystrophy mutations

**DOI:** 10.1007/s00415-025-12987-4

**Published:** 2025-03-04

**Authors:** Pietro Riguzzi, Daniele Sabbatini, Aurora Fusto, Sara Vianello, Beatrice Merlo, Vittoria Zangaro, Giuliana Capece, Domenico Gorgoglione, Gianni Sorarù, Riccardo Bariani, Chiara Calore, Barbara Bauce, Marika Martini, Anna Mutterle, Luca Bello, Elena Pegoraro

**Affiliations:** 1 Department of Neurosciences DNS, University of Padova, Via Giustiniani, 5, 35128 Padua, Italy; 2 Unit of Biostatistics, Epidemiology and Public Health, Department of Cardiac, Thoracic, Vascular Sciences, and Public Health, University of Padova, Padua, Italy; 3 Department of Cardiac, Thoracic, Vascular Sciences and Public Health, University of Padova, Padua, Italy

**Keywords:** Duchenne muscular dystrophy, Dystrophinopathies, Dystrophin, Dystrophinopathy carrier, Muscular dystrophies, Myopathies

## Abstract

**Objective:**

Duchenne muscular dystrophy (DMD) is an X-linked muscular dystrophy due to null mutations in the *DMD* gene that predominantly affects males, while heterozygous females are usually asymptomatic carriers. In approximately 10–20% of cases, they may present with muscle weakness and/or cardiomyopathy. We aimed to describe clinical and molecular characteristics of DMD heterozygous females.

**Methods:**

A monocentric, observational, and cross-sectional study was designed. Clinical and molecular data were collected along with, when available, muscle biopsies. The pattern of X inactivation was determined in peripheral blood and the genotypes at *SPP1*, *LTBP4* and *CD40* modifier genes were established.

**Results:**

We recruited 47 participants: 27 (57%) were asymptomatic and 20 (43%) manifested symptoms. Proximal muscles were prominently involved, as in male dystrophinopathies. Twenty % of carriers showed cardiac involvement. Creatine kinase (CK) values were in the normal range in ~ 20% of symptomatic and ~ 46% asymptomatic patients. In all muscle biopsies, a mosaic of dystrophin positive and negative fibers was observed that only marginally correlated to dystrophin amount. No correlation was found between X chromosome inactivation pattern and the severity of muscular involvement, nor any association with cardiomyopathy. No genotype–phenotype correlations were identified.

**Interpretation:**

Genotype/phenotype correlations in females heterozygous for *DMD* mutations are influenced by multiple mechanisms, of which better understanding will be crucial for future dystrophin gene replacement therapies. An earlier molecular identification is essential to lead to greater awareness of the potential cardiac complications, and hence the reinforcement of  appropriate cardiac follow-up.

**Supplementary Information:**

The online version contains supplementary material available at 10.1007/s00415-025-12987-4.

## Introduction

Duchenne muscular dystrophy (DMD) is a severe recessive X-linked muscle disease caused by mutations in the *DMD* gene, resulting in the absence in skeletal muscle of dystrophin, a protein essential to ensure structural integrity to muscle fibers. According to the Haldane theory, one-third of the cases are caused by de novo mutations [1, 2].

Females heterozygous for null *DMD* mutations are usually asymptomatic carriers, often presenting with high creatine kinase (CK) levels as the only clinical sign; however, despite the presence of a wild-type *DMD* allele, they can manifest skeletal muscle symptoms. These patients have been traditionally referred to as “manifesting carriers”; however, this terminology has recently been discouraged in preference of the clearer definition “females with dystrophinopathy”, leaving the term “carriers” to indicate only asymptomatic heterozygous females [3]. In females with dystrophinopathy the age of onset is variable, ranging from early childhood to late adulthood. Disease severity is also variable and genotype–phenotype correlations are not well established [4].

Females with *DMD* gene variations, even in the absence of skeletal muscle symptoms, have an estimated 7.3–16.7% lifetime risk of developing cardiomyopathy, characterised by left ventricular dilatation and decreased shortening fraction [5, 6]. Cardiac manifestations may be present without concomitant skeletal muscle involvement [7].

Several mechanisms have been hypothesized to explain clinical manifestations in female dystrophinopathy, and skewed X-chromosome inactivation (XCI) has been considered the main mechanism dictating the phenotype [8–10]. Nevertheless, studies on the role of XCI have highlighted discordant results: some researchers report a correlation between symptomatic female patients and skewed XCI [8–10], while others deny this correlation [11, 12]. Differences in the methodology of these studies make direct comparisons challenging. These differences include variations in the definition of symptoms, with some studies encompassing both skeletal muscle and cardiac involvement, the type of tissue used for studying X inactivation (e.g., lymphocytes vs. muscle tissue), the thresholds to define skewness, and the ability to identify the X chromosome carrying the mutated *DMD* gene.

Although an increasing number of studies are providing insights into the genetic and molecular basis of pathogenesis in female dystrophinopathy, the wide phenotypic variability observed in these individuals is still not well explained [4, 13]. Dystrophin levels in skeletal muscle may be a key factor in determining clinical severity. Additionally, biochemical and genetic normalization may be potential mechanisms that could further complicate the correlation between XCI and clinical severity [14]. Thus, more clinical studies are needed to establish robust genotype–phenotype correlations, which will eventually point to the amount of dystrophin needed to preclude clinical phenotype and to understand the extent to which mosaic muscle can behave as genetically and biochemically normal.

## Materials and methods

### Patients’ cohorts and inclusion criteria

An observational and cross-sectional study of the clinical and molecular characteristics of DMD heterozygous females was designed. We identified eligible subjects with a twofold strategy: first, the DNA and muscle biopsy biobank of the Neuromuscular Center of the University of Padova was screened to identify heterozygous females who had donated a sample; second, the database of DMD patients of the Neuromuscular Center was searched to identify heterozygous females among relatives of affected males. Individuals were further selected according to the following criteria: (1) molecular characterization of a heterozygous *DMD* mutation; (2) availability of a DNA sample; (3) available clinical data (neuromuscular evaluation, with or without cardiac evaluation). Clinical investigations adhered to the Declaration of Helsinki principles, and written informed consent, approved by institutional review boards, was obtained from all participants or their guardians in accordance with ethical requirements. The coordinating center IRB protocol number is 428n/AO/23.

### Clinical evaluation

Family and medical history were recorded for each participant. Disease endpoints (including age at loss of ambulation [LoA], age at loss of ability to rise from the floor, diagnosis of cardiomyopathy) were collected. All participants were evaluated in accordance with a standardized protocol that included anamnestic data and manual muscle strength testing in proximal and distal muscles of the upper and lower limbs and axial muscles (Medical Research Council [MRC] scale). For classification of disease severity, one proximal muscle of the upper (deltoid) and three muscles of the lower limbs (iliopsoas, gluteus maximus, and quadriceps) on both sides were considered. Levels of serum CK were measured (normal range 0–217 U/L, 97.5th percentile values for non-black females) [15].

To maintain consistency with definitions in the existing literature [9], participants were classified as: (1) asymptomatic carriers, if no muscle symptoms or signs were present or when only high CK levels and/or myalgias and/or calf hypertrophy were present, but without any muscle weakness; (2) mild symptomatic, when proximal muscle weakness was mild (MRC > 4 in at least one proximal muscle); (3) intermediate symptomatic with proximal muscle weakness between MRC grades 3 and 4 (in at least one proximal muscle); (4) severe symptomatic, with muscle weakness less than MRC grade 3 (in at least one proximal muscle) or loss of ambulation; (5) DMD-like, when characterised by LoA before the age of 12 years. Cardiomyopathy was not considered in this classification based on skeletal muscle function.

### Cardiac evaluation

Most participants underwent cardiological evaluation including clinical examination, electrocardiogram (ECG), 24-hour ECG Holter monitoring and 2D-Doppler echocardiogram. Clinically, the presence of signs and symptoms of heart failure was assessed.

The first cardiological evaluation was performed starting from late adolescence or upon the onset of symptoms suggestive of cardiac involvement. In case of normal findings, the cardiac assessment was repeated every 3 or 5 years, in accordance with international recommendations. In case of abnormal findings, the timing of the follow-up was dictated by cardiologists, typically ranging between 6 and 12 months [16, 17].

Twelve-lead ECGs were performed on a standard speed paper (25 mm/sec, 10 mm/mV, 0.05–150 Hz) at each evaluation. The following parameters were considered: type of rhythm (sinus rhythm, sinus arrhythmia, atrial fibrillation); the presence of atrioventricular block; fragmentation of QRS; complete right bundle branch block (RBBB): QRS duration > 120 ms, rSR’ in V1–V2 and deep and broadened S wave in V5–V6; complete left bundle branch block: QRS duration > 120 ms, deep S-wave in V1–V2 with small or absent initial r-wave, and widened R-wave with a notch in V5–V6, often also in DI and aVL, with absent q-wave; low voltages: defined as QRS of amplitude < 5 mm in peripheral leads or < 10 mm in precordial leads: repolarization abnormalities. Echocardiographic examinations were performed with a commercially available GE VIVID E9 ultrasound machine (GE Vingmed Ultrasound AS, Horten, Norway). Echocardiographic protocols and measurements were performed according to international recommendations [18]. Cardiomyopathy was defined in the presence of an indexed end diastolic volume of left ventricle (LV-EDVi) ≥ 70 ml/m^2^ and/or LV ejection fraction (LV-EF) ≤ 50% and/or wall motion abnormalities/segmental hypokinesia.

### Dystrophin western blot analysis

Fifteen patients underwent diagnostic muscle biopsy. Western blotting was done to evaluate dystrophin protein expression in extracts obtained from the biopsy samples. Dystrophin detection was performed using the mouse monoclonal antibody directed against the carboxyl terminus of dystrophin (NCL-DYS2, 1:800; Novocastra Laboratories Ltd, Newcastle, UK); samples were normalized using the mouse monoclonal anti-actin antibody (MAB1501, 1:5000, Millipore, Massachusetts, USA). Densitometric analysis of bands was performed with ImageJ software (National Institute of Health, USA) and dystrophin expression was normalized to β-actin.

### Dystrophin immunofluorescence staining

Cryostat sections were obtained from 12 muscle biopsies. The samples were incubated with mouse monoclonal antibody against the carboxyl terminus of dystrophin (NCL-DYS2, 1:100; Novocastra Laboratories Ltd, Newcastle, UK). After washing, specific labelling was developed by immunofluorescence, using anti-mouse Cy-3 conjugated immunoglobulin (1:100; Caltag, Burlingame, CA). Sections were mounted with anti-fading medium and examined with a video-confocal microscope (ViCo, Nikon Instruments). Serial cryosections were stained with haematoxylin–eosin to check tissue integrity and histopathology.

### X-chromosome inactivation studies

Genomic DNA was extracted from peripheral blood. We used the highly polymorphic CAG repeats in the first exon of the androgen receptor gene (*AR*) to identify the two X chromosomes in the women studied. Since it has been shown that the methylation status of the CAG repeats correlates with the extend of X-inactivation [19], we were able to identify the more active X chromosome. To do so, the DNA was digested with *HpaII* (R0171S, NEB) and *HhAI* (R0139S, NEB) methylation sensitive enzymes and purified by phenol/chloroform and ether extraction. An equal volume of non-digested DNA was processed as control. Seventy-five nanograms were used as templates for the amplification of the (CAG)n repeat in the first exon of the *AR* gene [9]. Sequences of the primers used are as follows: *AR* forward 5′- GCTGTGAAGGTTGCTGTTCCTCAT‐3′ and *AR* reverse 5′‐ATGAGGAACAGCAACCTTCACAGC‐3′. The digested and non-digested samples were run in parallel on ABI sequencer and peak heights and peak areas were analyzed using PeakScan software. The ratio between the two alleles of the undigested sample was calculated and the resulting correction factor was applied to allele values in the PCR amplified digested samples. The degree of X-inactivation was calculated by normalizing the sum of the two alleles in the digested sample to 100%. We defined skewed X-inactivation in peripheral blood DNA as a condition where one X chromosome is preferentially active in 70% or more of the nuclei. We analyzed the inheritance patterns of alleles by comparing PCR-amplified *AR* allele traces from the heterozygous females with those of affected relatives (heterozygous females), using fluorescent molecular weight markers serving as reference points for precise alignment. This approach enabled the identification of the X chromosome carrying the *DMD* mutation in six cases.

### SNPs genotyping

SNP genotypes were determined by TaqMan (Thermo Fisher Scientific) assays, at the known *DMD* modifier genes expressed in cardiac tissue: *SPP1* rs28357094 [20], *LTBP4* rs10880, rs2303729 and rs1131620 [21], *CD40* rs1883832 [22]. For *LTBP4*, both the effect of the rs10880 SNP and of the full VTTT/IAAM haplotype were considered. To test genotype/phenotype correlation, the published inheritance models (i.e., dominant, additive, or recessive) were used.

### Statistical analysis

We classified DMD heterozygous females into “skewed” or “random” X chromosome inactivation (XCI), including in the former group female subjects with an X chromosome active in 70% or more of nuclei. The categorical variables referring to muscular involvement were transformed into ordinal variables (asymptomatic = 0, mild = 1, intermediate = 2, severe = 3, DMD-like = 4) and ordinal regression model was used to analyze their correlation with CK levels. To evaluate significant interactions between XCI pattern and CK, LV-EDVi and LV-EF values, Wilcoxon rank sum test was used. This was also employed to statistically assess the differences in XCI percentages between asymptomatic and symptomatic carriers. Categorical variables such as “myopathic” vs. “non myopathic” and “cardiopathic” vs. “not cardiopathic” were compared according to XCI pattern using Fisher’s exact test. Spearman’s rank test was also used to analyze the correlation between age and EDVi and LV-EF. We performed Kaplan–Meier time-to-event analysis to assess the median time at onset of muscular symptoms with stratification based on the pattern of XCI (“skewed” vs. “random”); median age at event was compared between groups with a log-rank test. Fisher’s exact test was employed to analyze the association between type of *DMD* gene mutation and clinical severity at the most recent assessment.

Multiple linear-regression and Cox proportional hazards regression were used to model the effect of the known *DMD* modifiers on quantitative factors (CK, LV-EDVi and LV-EF values) and the X-inactivation pattern on the onset of symptoms, respectively.

Hazard ratios (HRs) were calculated for each quantitative covariate and each level of categorical covariates. Reference levels were assigned HRs of 1.0. Quantitative variables were summarised as mean ± standard deviation (SD). Analyses were carried out with R (R Foundation, version 4.1.3), with statistical significance set at *p* < 0.05 for all tests (Holm correction applied). Survival and survminer packages were used for Kaplan–Meier analysis [23, 24].

## Results

### Ascertainment and selection

One hundred and nine (109) women were identified by our combined search strategy. Among them, 72 (66%) resulted to be heterozygous for *DMD* mutations. Based on the availability of clinical and/or cardiological data and DNA samples, we selected 47 DMD heterozygous women for further studies. Among them, 38 subjects were unrelated while 9 were distributed across 4 families. None of the individuals included were of black ethnicity.

### *DMD* mutations

Heterozygous *DMD* gene mutations were identified in all females (Supplemental Table S1). As expected, single- or multi-exon out-of-frame deletions represented the majority of mutations (20/47; 43%), followed by nonsense mutations (11/47; 23%) and out-of-frame duplications (6/47; 13%). In the remaining patients, we identified small out-of-frame deletions (4/47; 8%), intronic mutations (5/47; 11%) predicting aberrant splicing with disruption of the open reading frame, and one large *DMD* gene rearrangement (1/47; 2%).

### DMD carriers’ cohort

Of the 47 females meeting our selection criteria, 27 (57%) were asymptomatic carriers (grade 0), while 20 (43%) exhibited symptoms of dystrophinopathy. According to phenotype classification among the symptomatic women, 7 showed a mild (grade 1), 6 an intermediate (grade 2), and 7 a severe phenotype (grade 3); none were DMD-like (grade 4). The global mean age at the last visit was 47.2 ± 14.4 years, 40.7 ± 10.4 years for the asymptomatic carriers, and 55.75 ± 14.4 years for the symptomatic participants (mild were 47.43 ± 15.27, intermediate were 57.80 ± 17.06, and severe DMD heterozygotes were 62.30 ± 6.52 years old). There was a significant correlation between age at the last visit and clinical severity (Spearman’s rank correlation rho = 0.63, *p* < 0.0001).

Clinical and genetic data are summarized in Supplemental Table S1. The age at last evaluation ranged from 14 to 80 years. Family history was positive in 38 (80%) cases. One patient with severe muscular weakness had died at the age of 65 years due to cardiac failure (#27).

Twenty (43%) heterozygous females exhibited muscle weakness (Supplemental Table S2). Age at symptom onset varied from 8 to 69 years (mean: 39.8 ± 16.7). In detail, mean age of onset was 32.9 ± 13.1 years in severely affected patients, 43.0 ± 21.0 in intermediate, and 44.0 ± 16.1 in mild patients. Proximal muscle weakness in the lower limbs (15/20; 75%) was the most common presenting symptom along with difficulty in running (4/20; 20%), jumping (1/20; 5%), or climbing stairs (3/20; 15%). Among first symptoms, myalgia was frequently reported (6/20; 30%), accompanied by other milder muscle symptoms such as subjective fatigue and/or muscle weakness. All patients but one (#3) were ambulant at last clinical evaluation and five (patients #5, #10, #11, #29 and #35) displayed severe impairment of ambulatory function, as they were only able to walk with support. Five of 7 severely affected women had lost the ability to rise from the floor. Asymmetry, defined as asymmetric muscle weakness upon physical examination, was observed in six patients (#5, #27, #29, #35, #36, #45). Of these individuals, four exhibited greater weakness on the right side and two on the left.

Gluteus, iliopsoas, quadriceps femoris, and deltoid were the most prominently affected muscles, as expected in dystrophinopathies. However, in our cohort, distal weakness was often observed, especially in severe (6/7) and intermediate (3/6) patients. Axial muscles were also frequently involved (12/20). Macroglossia was observed in four symptomatic patients and in one who was asymptomatic. Calf hypertrophy was a frequent clinical feature both in symptomatic (11/20; 55%) and asymptomatic women (6/27, 22%).

**Fig. 1:**
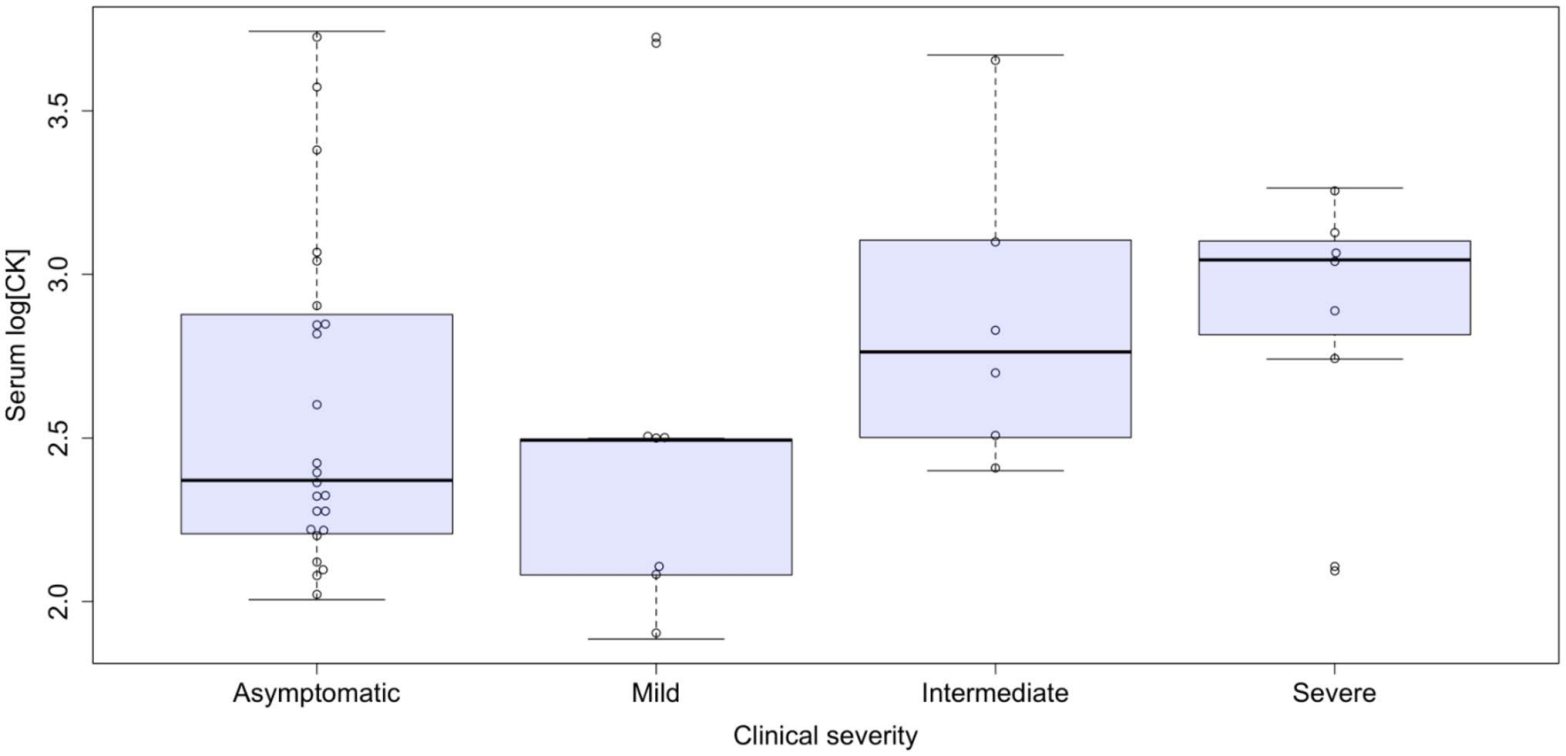
Serum CK levels (UI/L, log base 10) according to skeletal muscle phenotypes. Ordinal logistic regression: *p* = n.s

CK levels were available in 44 participants. High serum CK levels (defined as above 217 U/L) were detected in 13 (13/24; 54%) asymptomatic carriers and 16 (16/20; 80%) symptomatic patients (Supplemental Table S1), with mean values of 812 ± 1275 U/L and 1037 ± 1378 U/L, respectively (*p* = n.s.). An ordinal regression analysis for CK levels did not reveal a statistically significant association with clinical severity (Fig. 1).

### Cardiac function

Cardiac data was available in 44/47 patients and is summarized in Supplemental Table S3. Cardiac involvement in terms of LV dilatation/dysfunction was found in 9 women: 3/7 (43%) of severely, 3/6 (50%) of intermediate, and 2/7 (29%) of mildly affected patients, and in 1/23 (4%) of the asymptomatic carriers. The mean age at detection of cardiac involvement was 56.2 ± 14.9 years (min 32, max 74 years). Thirteen patients (28%) received a cardiological therapy, including angiotensin-converting enzyme inhibitors (ACEi), beta blockers (BB), diuretics, calcium channel blockers (CCB), angiotensin receptor blockers (ARB) and anticoagulants, most often as combination therapy that included two or more medications. Nine out of these had a cardiomyopathy, while subjects #2, #7, #35 and #36 were only treated for high blood pressure.

By means of echocardiographic analysis, a LV dilatation was detected in five (5/44, 11%) patients (mean total cohort EDVi 54.5 ± 15.5 ml/m^2^, range 33–122 ml/m^2^), while six subjects (6/44, 13.6%) exhibited a LV-systolic dysfunction (mean total cohort LV-EF 58.4 ± 8.7%, range 32-78%; Supplemental Figure S1 and S2; Supplemental Table S3). There was a significant negative correlation between age and LV-EF (Spearman’s rank test rho = − 0.35, *p* = 0.02), while no correlation was found with EDVi (rho = − 0.02, *p* = 0.9). In 9/40 (22.5%) cases wall motion alterations (WMA) were detected, mainly in basal segments of posterior and inferior walls. Concerning the right ventricle (RV), a dilation was observed in 2 patients (2/44, 5%; mean end-diastolic area [RV-EDAi] of 8.88 ± 2.39 cmq/m^2^, range 6–19 cmq/m^2^), while a dysfunction in one case (1/44, 2%; mean fractional area change [FAC] of 47.6 ± 12.2%). None presented with RV-WMA. Fifteen patients (5 symptomatic and 10 asymptomatic) underwent myocardial strain analysis, and abnormal values were detected in two symptomatic (2/5; 40%) and in two asymptomatic women (2/10; 20%). Given the known association between high blood pressure and impaired global myocardial systolic strain, we ruled out hypertension in three of the four patients with strain abnormalities.

ECG was available in 42 patients (89%). All had sinus rhythm except one with persistent atrial fibrillation. Two patients had intraventricular conduction delay (incomplete right bundle branch block and anterior fascicular block, respectively). No patients had ventricular repolarization abnormalities. On 24-hour Holter ECG monitoring, three patients showed supraventricular ectopic beats, and another three showed premature ventricular beats. Moreover, two patients required implantable cardioverter defibrillator (ICD) implantation. More specifically, patient #21 was implanted in secondary prevention due to an episode of sustained ventricular tachycardia (SVT), whereas patient #27 was implanted in primary prevention at the age of 63, two years before her death for heart failure due to dilated cardiomyopathy with a severe reduction of LV systolic function.

Symptoms of heart failure were rare, only reported by these two patients with severe cardiomyopathy (#21, #27).

### Dystrophin protein studies

In our cohort, six asymptomatic carriers and nine symptomatic patients underwent a diagnostic muscle biopsy; in asymptomatic carriers, the indication was elevated CK. All biopsies were analyzed by dystrophin immunoblotting assay, and 12 also by immunofluorescence analysis.

### Dystrophin immunofluorescence

**Fig. 2:**
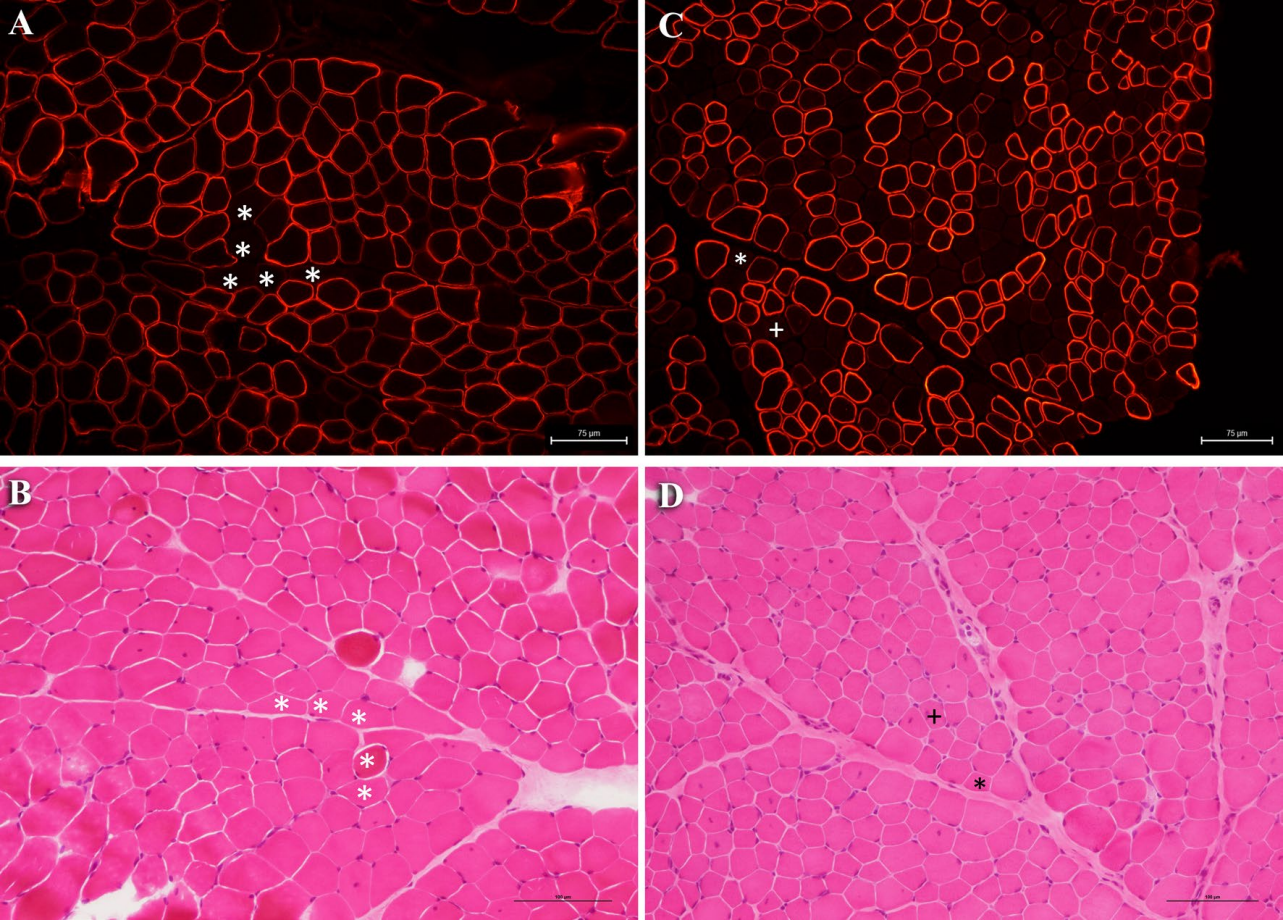
Immunofluorescence analysis shows a mosaic pattern of dystrophin positive and negative fibers in DMD heterozygous females. Cryosections were stained with anti-dystrophin antibody NCL-DYS2 (panel **A** and **C**) and, as an example, some dystrophin negative fibers are indicated by an asterisk. **A** Immunolabeling of a 4-year-old girl (patient #43) and **C** of a 2-year-old girl (patient #9) showing dystrophin negative fibers. Scale bar: 75 μm. In panels **B** and **D** serial sections are stained with haematoxylin and eosin staining to check tissue integrity and histopathology. Scale bar: 100 μm

All biopsied patients showed a mosaic of dystrophin positive and negative fibers (Fig. 2). All muscle fibers in muscle cryosections stained with an anti-dystrophin antibody were counted on photographic enlargement and used to calculate the percentage of dystrophin positive and negative fibers (Supplemental Table S4). The mean percentage of dystrophin negative fibers was 32 ± 41.3 in symptomatic patients, and 11.5 ± 2.9 in asymptomatic carriers.

### Dystrophin immunoblotting

**Fig. 3:**
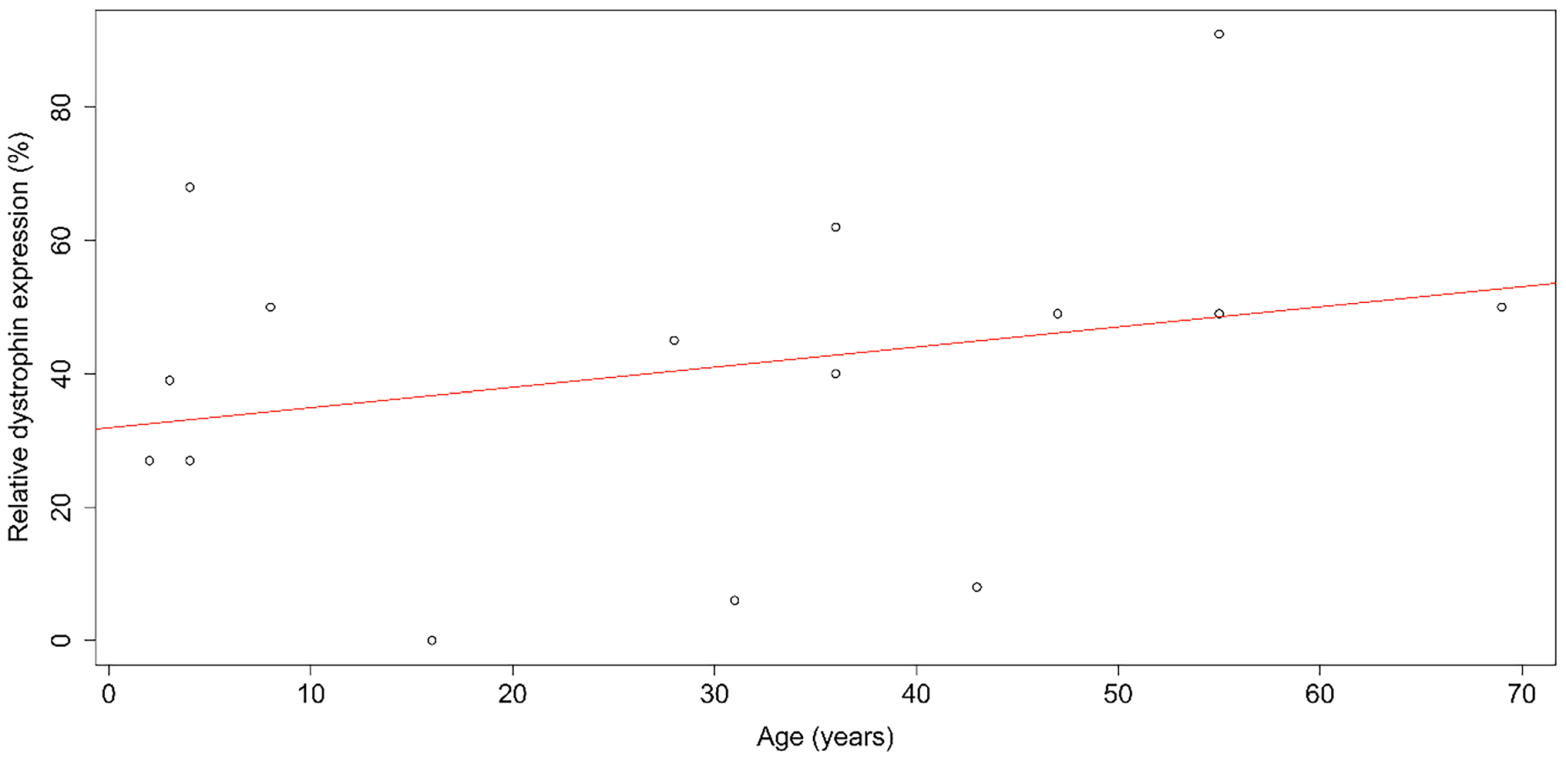
Scatter plot of dystrophin amount by age at muscle biopsy. Spearman’s rank test: rho = 0.34, *p* = 0.20

In all muscle biopsies, normal molecular weight but reduced amount of dystrophin was detected. Dystrophin levels were 49.2% ± 14.9% of those observed in healthy controls in the asymptomatic carriers, and 35.1% ± 28.6% in symptomatic patients (*p* = n.s.). No correlations between the amount of dystrophin, age at muscle biopsy (Fig. 3) and age at onset of muscular symptoms were observed.

### XCI pattern in blood DNA

Eighty-five percent (40/47) of participants were heterozygous at the *AR* locus, and the XCI pattern was assessed (Supplemental Table S4). Nine symptomatic DMD heterozygous females showed a random XCI pattern (9/18, 50%), and nine a skewed pattern (9/18, 50%). Among asymptomatic subjects, 8 showed random X inactivation (8/22, 36.5%), and 14 skewed X inactivation (14/22, 63.5%).

In six females, the segregation analysis of the *DMD* mutation in affected family members allowed for the identification of the X chromosome carrying the mutated *DMD* gene. This was possible when a DMD heterozygous female relative was available, and the phase between the *AR* and the *DMD* gene was clearly established. Among these six individuals, three were symptomatic, and in all the X inactivation favored the normal X chromosome, although the degree of inactivation was extremely variable, with only one individual showing a skewed XCI (#29) pattern, thus resulting in the severe symptomatic phenotype observed. The remaining three female carriers were asymptomatic with a random pattern of XCI.

From the ordinal regression analysis, the estimated effect of XCI on clinical severity showed a coefficient of 0.4505 suggesting that XCI does not predict clinical severity. Moreover, the Wald test did not indicate a statistically significant association (χ^2^ = 1.97, *p* = 0.16).

No significant correlation was observed between XCI pattern and cardiac involvement, nor skeletal muscle involvement (Fisher’s exact test *p* = n.s.).

Median CK values were 316.5 U/L in “random” and 400 U/L in “skewed” DMD heterozygous females (Wilcoxon rank-sum test *p* = 0.95, Supplemental Figure S3A).

The comparison of LV-EDVi between groups with random and skewed XCI (Supplemental Figure S3B) was assessed using a Wilcoxon rank-sum test. The median EDVi was 54 ml/m^2^ for the random and 50.5 ml/m^2^ for the skewed XCI groups (*p* = 0.55). Similarly, the comparison of LV-EF% between groups was not significant (*p* = 0.37), with median EF of 58% for the random XCI group and 60% for the skewed XCI group (Supplemental Figure S3C).

**Fig. 4:**
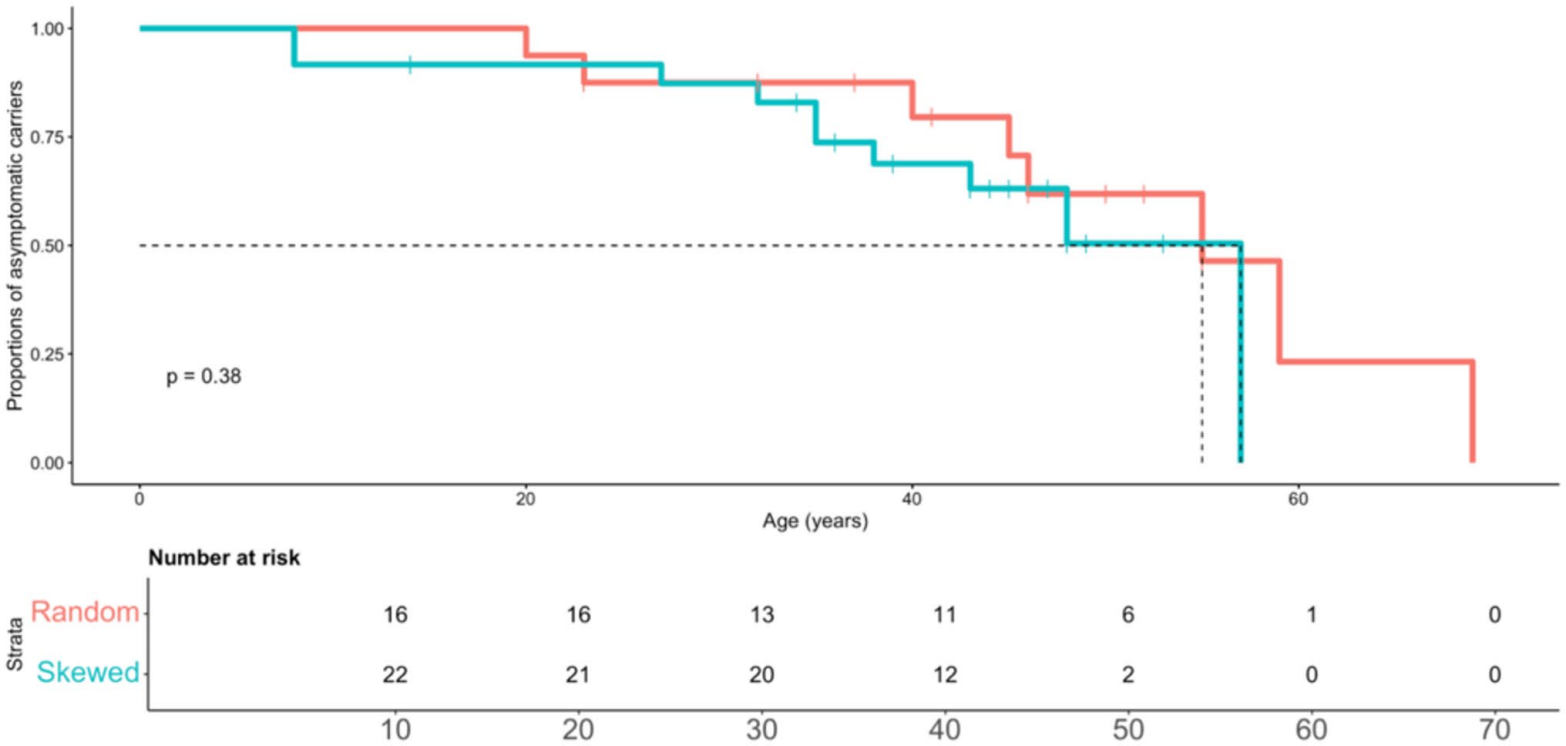
Kaplan–Meier analysis comparing pattern of XCI (“random” vs. “skewed”) to age of onset of clinical manifestations in skeletal muscle of DMD heterozygous females.

A Kaplan–Meier time-to-event analysis was performed to assess the median time of the onset of muscular symptoms with stratification based on the pattern of XCI (“skewed” vs. “random”). The median age at onset was 55 years for the random XCI group and 57 years for the skewed XCI group, with no statistically significant difference between the two groups (log-rank test, *p* = 0.38) (Fig. 4).

The median percentage of XCI was 74.5% for the asymptomatic patients and 73.5% for the symptomatic ones, with no statistically significant differences between groups (Wilcoxon rank-sum test *p* = 0.6, Supplemental Figure S4).

### Modifier genotypes

*DMD* gene mutations were classified in deletions, duplications, nonsense, and intronic. The Fisher’s exact test revealed no statistically significant difference in the distribution of phenotypic severity across the various groups of *DMD* mutation types in our dataset (*p* = 0.56).

Genotype analysis of the modifier SNPs in *SPP1*, *LTBP4* and *CD40* genes was performed in 45 subjects (18 symptomatic patients and 27 asymptomatic carriers). We used a Cox regression model to estimate the effect of variants in *SPP1*, *LTBP4* and *CD40* genes on the onset of symptoms and a multiple linear regression model to evaluate the effect of the SNPs on quantitative factors (CK, LV-EDVi and LV-EF values). No correlation between the genotypes and any of the variables considered was observed.

## Discussion

In order to shed light on the mechanisms responsible for the clinical manifestations of dystrophinopathy in females, we recruited a cohort of 47 DMD heterozygous females, 27 asymptomatic and 20 symptomatic. While this sample size may not be considered large in absolute terms, both the rarity of the disease and the monocentric design have to be taken into consideration. The identified rate of 42% symptomatic patients is high compared to the rates reported in the literature [25]. This may be due to a “selection bias”, as several carriers were recruited because they presented with muscle weakness and had undergone a muscle biopsy for diagnostic purposes.

Consistent with previous reports [4, 26], the spectrum of clinical manifestations in symptomatic patients was wide. Loss of independent ambulation appears to be a relatively rare event in females with dystrophinopathy, since in our cohort, all patients but one were ambulant at the last clinical evaluation, and none had a DMD-like phenotype. However, five displayed severe impairment of ambulation, as they were only able to walk with support, and six patients had lost the ability to rise from the floor independently, which is a predictor of potential loss of ambulation. The pattern of muscle weakness is similar to that observed in male dystrophinopathy, with proximal muscles being more frequently involved and distal muscle weakness exclusively present in severe or moderately weak patients.

We confirmed a particular clinical characteristic of symptomatic patients, i.e., potential asymmetry of muscle weakness and wasting. Asymmetry in muscle involvement was observed in 30% of cases, and it was particularly pronounced in one patient (#29, skewed XCI pattern); however, no consistent preference for one side was noted. Asymmetry represents an important diagnostic clue that should orient clinicians towards a suspicion of dystrophinopathy in females. The pathophysiology of this phenomenon probably resides in lateralized clonal expansion of embryonic precursor cell pools with skewed X inactivation, as indirectly confirmed by findings in cases of somatic *DMD* mutations in male “mosaic” patients, who may also show asymmetric involvement [27, 28].

In this study, as expected, raised serum CK levels were found in the majority of female patients, as only 20% of symptomatic patients and 46% of asymptomatic carriers had normal values (< 217 U/L) [29]. Therefore, elevated CK represents an important sign of muscle involvement in the diagnostic phase, especially when a family history is not available. On the other hand, normal levels cannot rule out a heterozygous *DMD* mutation, even in patients with muscle weakness. The magnitude of CK elevation did not correlate with the severity of muscle involvement, with severely affected patients showing higher CK levels on average; however, very high CK values (more than 1000 U/L) were also observed in some asymptomatic carriers.

In our cohort, nine women had no family history of dystrophinopathy, posing a diagnostic challenge. This absence may be due to a genuine lack of other affected family members, possibly resulting from a de novo mutation in the paternal [9] or maternal lineage. Alternatively, it could be attributed to potential mosaicism in ancestors, which could have attenuated the severity of the phenotype, preventing clinical detection. In DMD heterozygous females, mosaicism may also be present, with different ratios of X-inactivation across different tissues, potentially mitigating or exacerbating the severity of the phenotype.

Clinical severity was found to correlate with age, underscoring the progressive nature of muscle involvement in female patients with dystrophinopathy. However, no such correlation was observed with the XCI inactivation pattern. This lack of association may stem from the inability to identify the X chromosome carrying the *DMD* mutation in most patients. Despite this, in six patients, we were able to accurately determine the phase of the X chromosome harboring the mutation. This was possible in three daughters (#39, #40, #43) of heterozygous mothers (#47, #27, #35) and a family group composed of a daughter (proband), her mother, and her aunt (#30, #28, #29). Among these cases, one patient exhibited a “skewed” X inactivation pattern and had a severe phenotype, while the others displayed a “random” inactivation pattern, with either mild weakness or an asymptomatic presentation, further supporting the role of the XCI pattern in influencing clinical severity.

The discordant results about the role of XCI in females with dystrophinopathy in the literature are likely due to different factors. Firstly, as in our study, where the linkage phase cannot be determined, i.e. where it is unknown which X chromosome harbors the *DMD* mutation, it is not possible to establish whether the pattern of XCI favors the normal or mutated X chromosome. Secondly, in the literature, the classic definition of “manifesting DMD carrier” has included both patients presenting with muscle weakness and/or dilated cardiomyopathy [11, 12], whereas cardiac and skeletal muscle involvement can occur independently. Finally, the pattern of XCI as ascertained from lymphocyte DNA may not reflect the XCI pattern in skeletal muscle in any individual patient.

Dystrophin levels were only available in a minority of patients, and no clear correlation with levels of dystrophin and age at muscle biopsy was found. Dystrophin immunofluorescence, on the other hand, proved to be a sensitive test to identify DMD heterozygous females. All the biopsied women in our cohort showed a mosaic of dystrophin positive and negative fibers that only marginally correlated to dystrophin amount by Western blot. Indeed, less than 20% of dystrophin negative fibers allowed the production of 27% to 91% of normal dystrophin as assessed by Western blot. Unfortunately, the amount of dystrophin did not correlate with clinical muscle involvement, age at biopsy or age at onset of symptoms. Even if it is reasonable to assume that, in young females, dystrophin amount may be dictated only by the pattern of X inactivation prior to the normalization phenomena that may appear later, it is hard to predict the efficiency in phenotype rescue and the timing of the normalization phenomena [14].

It is important to acknowledge that studying the correlation of dystrophin levels with age may be influenced by a selection bias in cross-sectional studies. Patients who undergo a muscle biopsy at an older age are likely to have a later onset of symptoms, thus to be milder a priori. Studies on dystrophin expression on longitudinal muscle biopsies in heterozygous females with *DMD* mutations could help address some questions as to the extent to which the normalization phenomena have an impact on the clinical severity and contribute to phenotype rescue.

Although the majority of DMD heterozygotes do not exhibit skeletal muscle weakness, they may develop cardiac involvement. Cardiac manifestations encompass a wide spectrum of variability, ranging from mild conduction defects and heart rhythm abnormalities to dilated cardiomyopathy and progressive heart failure [5, 7], requiring cardiac transplantation in extreme cases [30, 31]. In our cohort, cardiac involvement was detected in 20% of female patients, which is comparable with previous reported data [5]. The most commonly observed phenotype was dilated cardiomyopathy, in the presence of WMA. In our cohort, one of nine cardiomyopathy patients did not show any skeletal muscle weakness (asymptomatic) and a carrier status diagnosis was prompted by a positive family history. Only after the DMD carrier diagnosis, the cardiac surveillance protocol was applied, allowing an early diagnosis in a pre-symptomatic stage of heart involvement. Hence, it is crucial to increase awareness of a possible cardiomyopathy in DMD carriers, even if asymptomatic. It may also be prudent to consider conducting multiplex ligation probe amplification (MLPA) assay for the *DMD* gene in female patients with dilated cardiomyopathy, irrespective of the absence of overt muscle weakness, high CK levels, or family history of dystrophinopathy. It has been hypothesised that in females with heterozygous *DMD* mutations the onset of cardiomyopathy may be associated with skewed XCI, as in skeletal muscle [10]. It is reasonable to state that peripheral blood DNA X inactivation pattern may not accurately predict a cardiac phenotype. There are several reasons for this discrepancy. Firstly, the repeated cycles of degeneration and regeneration that occur in skeletal muscle promoting both genetic and biochemical normalization (i.e. increase amount of dystrophin with age) [14] are not present in the heart, where the absence of regeneration would prevent the partial restoration of dystrophin once degeneration occurs [30, 32]. Secondly, it is possible to speculate that the pattern of XCI may differ between tissues resulting in a different ratio of dystrophin-negative and positive fibers [33].

As anticipated due to the small cohort size, no correlation was found between the type of *DMD* mutation and clinical severity at the last assessment.

We also wanted to test whether the genetic modifiers involved in the modulation of age at loss of ambulation and cardiac phenotype in boys with DMD, might have a role in disease severity in females with dystrophinopathy.

We used a multiple regression-model to estimate the possible effect of variants in *SPP1*, *LTBP4* and *CD40* genes on the onset of muscle symptoms and CK levels, LV-EDVi and LV-EF values, but no significant correlations were found. Based on this preliminary study, we cannot rule out the role of these genetic modifiers, but probably both more robust outcome measures, which are lacking in females with dystrophinopathy, and much larger cohorts are needed.

This study is subject to the inherent limitations of retrospective cross-sectional designs, including potential recall and selection biases.

However, our observations carry relevant implications.

At the biochemical level, the heterozygous female population may provide valuable insights into the amount of dystrophin required to preclude clinical phenotype, and into the extent to which mosaic muscle can normalize genetically and biochemically. These findings could serve as crucial background data for future dystrophin gene replacement therapies, although larger cohorts are needed to achieve significant results.

In clinical practice, early identification of carriers is essential for increasing awareness of potential cardiac complications and ensuring appropriate cardiac surveillance. Additionally, timely detection is crucial for informing patients about their genetic recurrence risk.

Identifying DMD heterozygous females can be challenging due to the significant phenotypic variability. Some patients may not exhibit muscle weakness or elevated CK levels, and they may not experience cardiac symptoms, especially in the initial stages of cardiomyopathy. Therefore, when confronted with nonspecific symptoms such as myalgia, clinicians should remain vigilant for subtle signs of dystrophinopathy, such as macroglossia and calf hypertrophy, rather than relying solely on CK levels. Notably, applying the latest recommendations for asymptomatic/paucisymptomatic hyperCKemia, which suggest genetic testing for females with CK levels exceeding 1.5 times the upper limit of normal (325 U/L for non-black females), would have resulted in missed diagnoses for 58% of our asymptomatic patients (14 out of 24 with available CK values) had they not had a supportive family history [34].

## Supplementary Information

Below is the link to the electronic supplementary material.

Supplementary file1 (PDF 547 KB)

## Data Availability

Not applicable, all data is available in the supplementary materials. If additional data is required, the corresponding author will evaluate specific requests.
