# Supplementary material for: Deep characterization of females with heterozygous Duchenne muscular dystrophy mutations
Source: J Neurol. 2025 Mar 4;272(3):244. doi: 10.1007/s00415-025-12987-4 (PMC11880153; doi:10.1007/s00415-025-12987-4)
Supplement: Supplementary file 1 — Supplementary file1 (PDF 547 KB) [file 415_2025_12987_MOESM1_ESM.pdf]

Supplementary Materials

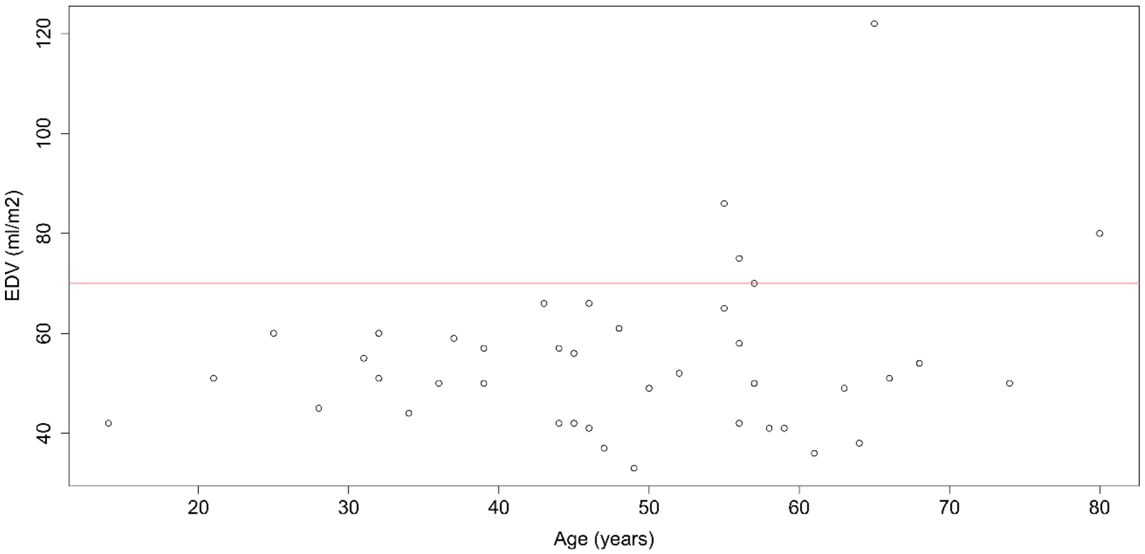

Figure S1. Scatter plot of left ventricular EDVi (ml/m2) by age. The pathological threshold is marked by a red line. Spearman's rank test: rho=-0.02, *p*=0.9.

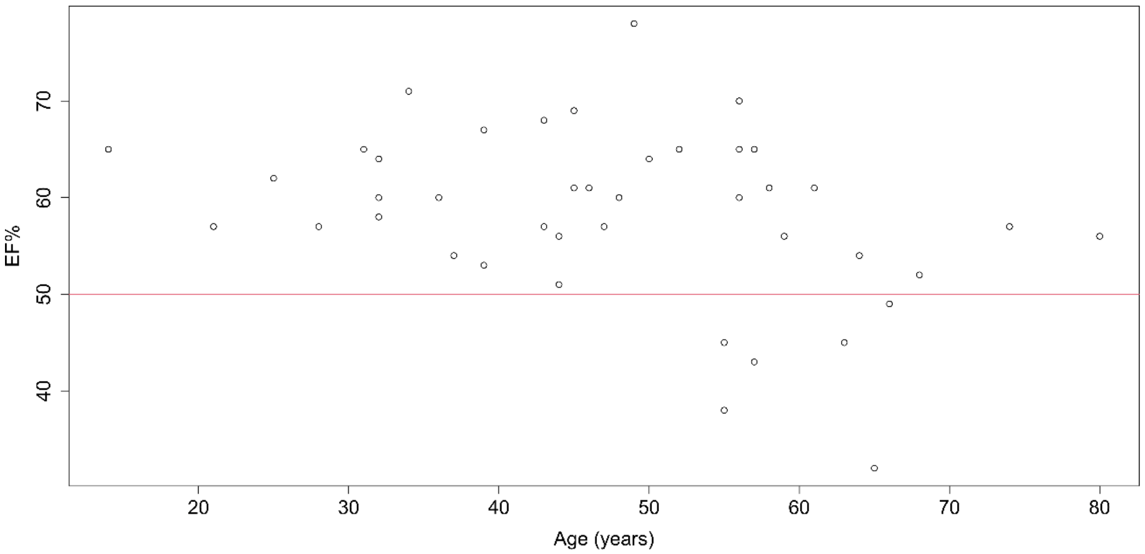

Figure S2. Scatter plot of left ventricular EF (%) by age. The pathological threshold is marked by a red line. Spearman's rank test: rho=-0.35, p=0.02.

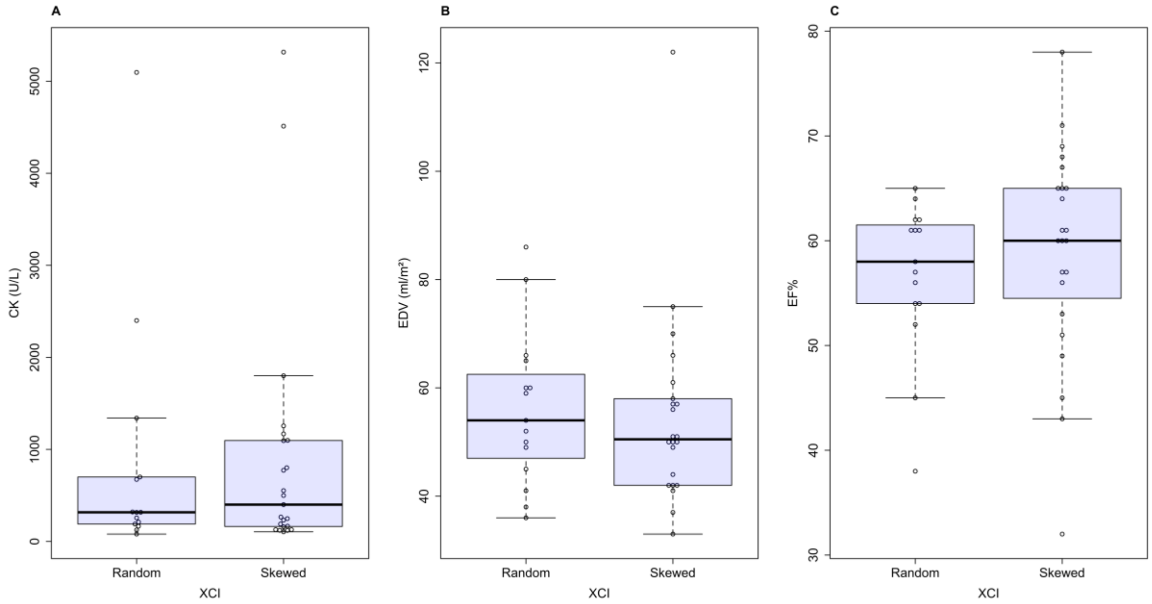

Figure S3. Beeswarm plot of serum CK levels (A), left ventricular EDVi (ml/m^2) (B) and EF (%) (C) in “skewed” vs. “random” DMD heterozygous females. Wilcoxon rank sum test with continuity correction: A) p=0.95; B) p=0.55, C) p=0.37.

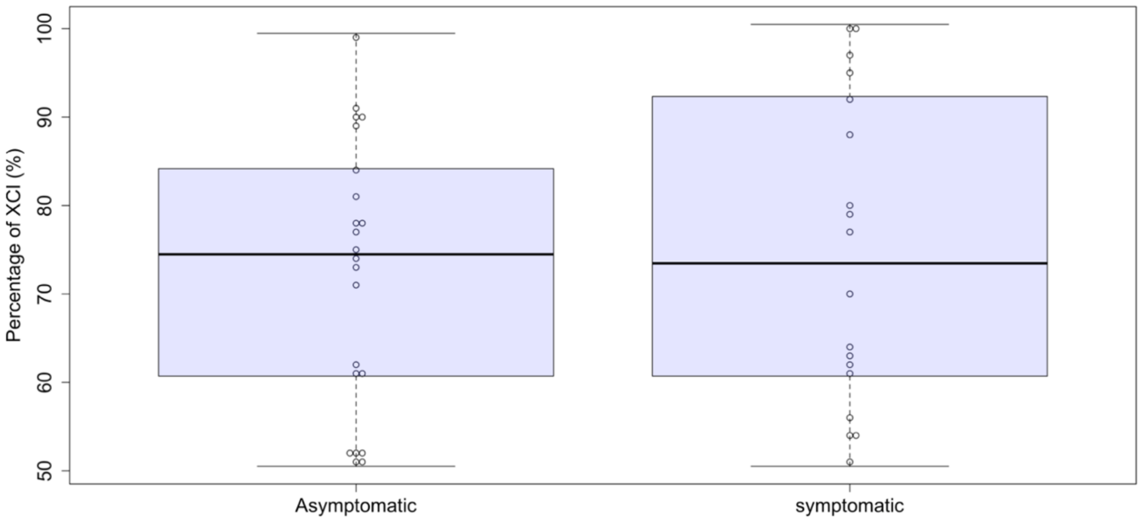

Figure S4. Beeswarm plot of XCI pattern (%) in Asymptomatic and Symptomatic patients. Wilcoxon rank-sum test, *p*=0.6.

Table S1. Clinical and genetic features of DMD heterozygous females.

ID	FAMILY	*DMD* MUTATION(S)	FAMILY HISTORY	CLINICAL SEVERITY	AGE AT ONSET	AGE AT LOA	AGE AT LAST VISIT	CARDIO-MYOPATHY	CK (U/L)
**1**	1	del Dp427c-45	Yes	Asymptomatic	-	Ambulant	43	No	801
**2**	2	dup 3-43	Yes	Mild	57	Ambulant	58	No	128
**3**	3	del 51	Yes	Severe	8	43	57	No	128
**4**	4	del 44	No	Intermediate	58	Ambulant	59	No	322
**5**	5	dup 28-43	No	Severe	35	Ambulant	66	Yes	773
**6**	6	c.2548G>T	Yes	Asymptomatic	-	Ambulant	45	No	105
**7**	7	c.2302C>T	Yes	Severe	50	Ambulant	74	No	1162
**8**	8	c.2656C>T	Yes	Asymptomatic	-	Ambulant	34	No	1168
**9**	9	del 10-21	Yes	Intermediate	8	Ambulant	32	No	499
**10**	10	c.7222_7223del	Yes	Intermediate	69	Ambulant	80	Yes	674
**11**	11	c.2548G>T	Yes	Intermediate	45	Ambulant	68	Yes	256
**12**	12	del 8-43	Yes	Asymptomatic	-	Ambulant	32	No	125
**13**	13	c.2555-2556delG	Yes	Asymptomatic	-	Ambulant	44	No	231
**14**	14	c.2285A>C; c.4495C>T	Yes	Asymptomatic	-	Ambulant	34	NA	265
**15**	15	c.8879G>A	Yes	Mild	46	Ambulant	46	No	316
**16**	16	c.6805C>T	Yes	Asymptomatic	-	Ambulant	55	Yes	700

**17**	17	del 52	Yes	Asymptomatic	-	Ambulant	39	No	248
**18**	18	c.1990C>T	Yes	Asymptomatic	-	Ambulant	39	No	189
**19**	19	del 49-52	No	Asymptomatic	-	Ambulant	43	No	1098
**20**	20	del 8-9	Yes	Asymptomatic	-	Ambulant	50	No	189
**21**	21	dup 6-7	Yes	Mild	55	Ambulant	55	Yes	320
**22**	22	del 44-47	Yes	Asymptomatic	-	Ambulant	44	No	159
**23**	23	c.9685delT	Yes	Asymptomatic	-	Ambulant	52	No	NA
**24**	24	dup 3-7	No	Asymptomatic	-	Ambulant	31	No	3740
**25**	25	c.3151C>T	Yes	Asymptomatic	-	Ambulant	48	No	166
**26**	26	del 61-63	Yes	Asymptomatic	-	Ambulant	53	NA	NA
**27**	27	del 49-54	Yes	Severe	38	Ambulant	65#	Yes	552
**28**	28	del 49-50	Yes	Mild	59	Ambulant	64	No	80
**29**	28	del 49-50	Yes	Severe	27	Ambulant	57	Yes	1095
**30**	28	del 49-50	Yes	Asymptomatic	-	Ambulant	37	No	211
**31**	29	del 45-52	Yes	Mild	48	Ambulant	56	Yes	121
**32**	30	c.1150-1G>T	Yes	Asymptomatic	-	Ambulant	41	No	NA
**33**	31	c.2168+1G>A	Yes	Asymptomatic	-	Ambulant	49	No	120
**34**	32	c.6591delA	No	Mild	20	Ambulant	28	No	5096
**35**	33	c.1507G>T	No	Severe	32	Ambulant	56	No	1800
**36**	34	del 46-55	Yes	Severe	40	Ambulant	61	No	1340

**37**	35	del 10-11	No	Asymptomatic	-	Ambulant	21	No	657
**38**	36	15 Mb inversion in g Xp21.1	Yes	Asymptomatic	-	Ambulant	47	No	400
**39**	37	dup 51-67	Yes	Mild	23	Ambulant	25	No	317
**40**	27	del 49-54	Yes	Asymptomatic	-	Ambulant	46	No	165
**41**	38	c.7310-19A>G	No	Asymptomatic	-	Ambulant	14	No	5316
**42**	39	del Dp427m-47	Yes	Asymptomatic	-	Ambulant	45	NA	132
**43**	33	c.1507G>T	Yes	Asymptomatic	-	Ambulant	23	NA	2400
**44**	40	c.358-2A>G	Yes	Asymptomatic	-	Ambulant	56	No	704
**45**	41	del 45-50	No	Intermediate	35	Ambulant	45	No	4512
**46**	42	c.2950-2A>C	Yes	Asymptomatic	-	Ambulant	32	No	210
**47**	37	dup 51-67	Yes	Intermediate	43	Ambulant	63	Yes	1255

LoA: loss of ambulation; NA: not available; del: deletion; dup: duplication.

Table S2. Clinical features in females with dystrophinopathy (manifesting DMD carriers).

ID	Clinical severity	Age at onset	Symptoms at onset	Age at LoA	Age at last visit	Muscle hypotrophy or hypertrophy	Gowers' manoeuvre	Macroglossia	Muscle weakness pattern	Right iliopsoas	Left iliopsoas	Right gluteus maximus	Left gluteus maximus	Right quadriceps	Left quadriceps	Right deltoid	Left deltoid
**2**	Mild	57	Myalgia, mild proximal muscle weakness in the lower limbs	Ambulant	58	No	No	No	Proximal; axial	5/5	5/5	4+/5	4+/5	5/5	5/5	5/5	5/5
**3**	Severe	8	Difficulty in running	43	57	Global muscle hypotrophy but mild calf hypertrophy	Unable to rise	No	Proximal> distal; axial	2/5	2/5	2/5	2/5	2/5	2/5	2/5	2/5
**4**	Intermediate	58	Myalgia, fatigue	Ambulant	59	Mild left calf hypertrophy	No	No	Proximal; Axial	4/5	4/5	4/5	4/5	4/5	4/5	5/5	5/5
**5**	Severe	35	Proximal muscle weakness	Ambulant	66	No	Unable to rise	No	Asymmetric; Proximal > distal; axial	5-/5	5/5	3-/5	3-/5	5/5	5-/5	4/5	5-/5
**7**	Severe	50	Difficulty in running	Ambulant	74	No	Yes	No	Proximal > distal; axial	4-/5	4-/5	2/5	2/5	3-/5	3-/5	5/5	5/5
**9**	Intermediate	8	Difficulty in running	Ambulant	32	Calf hypertrophy	Yes	No	Proximal	4/5	4/5	3/5	3/5	5-/5	5-/5	5-/5	5-/5
**10**	Intermediate	69	Difficulty in climbing stairs, myalgia	Ambulant	80	Calf hypertrophy	Unable to rise	Yes	Proximal > distal; axial	4+/5	4+/5	4-/5	4-/5	5/5	5/5	5/5	5/5
**11**	Intermediate	45	Proximal muscle weakness	Ambulant	68	Lower limb hypertrophy	Yes	No	Proximal>distal; axial	4-/5	4-/5	4-/5	4-/5	3/5	3/5	5/5	5/5
**15**	Mild	46	Myalgia, mild proximal muscle weakness in the lower limbs	Ambulant	46	No	No	No	Axial; proximal	5/5	5/5	5-/5	5-/5	5/5	5/5	5/5	5/5

**21**	Mild	55	Proximal muscle weakness	Ambulant	55	No	No	No	Proximal	5/5	5/5	4+/5	4+/5	5/5	5/5	5/5	5/5
**27**	Severe	38	Proximal muscle weakness	Ambulant	65	Calf hypertrophy	Unable to rise	Yes	Asymmetric; proximal> distal; axial	3-/5	4-/5	NA	NA	3-/5	3-/5	2/5	2/5
**28**	Mild	59	Myalgia, mild proximal muscle weakness in the lower limbs	Ambulant	64	No	Yes	No	Proximal	5/5	5/5	4+/5	4+/5	5/5	5/5	5/5	5/5
**29**	Severe	27	Proximal muscle weakness	Ambulant	57	No	Unable to rise	Yes	Asymmetric; proximal=distal	4/5	3-/5	4+/5	3-/5	4+/5	2/5	4/5	4/5
**31**	Mild	48	Proximal muscle weakness	Ambulant	56	No	Yes	No	Proximal	5/5	5/5	4+/5	4+/5	5/5	5/5	5/5	5/5
**34**	Mild	20	Difficulty in climbing stairs; frequent falls	Ambulant	28	No	No	No	Proximal	5-/5	5-/5	5/5	5/5	5/5	5/5	5/5	5/5
**35**	Severe	32	Difficulty in climbing stairs	Ambulant	56	Calf hypertrophy	Unable to rise	Yes	Asymmetric; Proximal>distal	3/5	3/5	2/5	2/5	3-/5	4/5	4/5	4/5
**36**	Severe	40	Proximal muscle weakness	Ambulant	61	Calf hypertrophy	Yes	No	Asymmetric; proximal	4+/5	3+/5	3+/5	2/5	5/5	3-/5	5/5	5/5
**39**	Mild	23	Myalgia, mild proximal muscle weakness in the lower limbs	Ambulant	25	Calf hypertrophy	No	No	Proximal; axial	4+/5	4+/5	4+/5	4+/5	5/5	5/5	5/5	5/5
**45**	Intermediate	35	Proximal muscle weakness	Ambulant	45	Left arm hypotrophy, right calf hypertrophy	Yes	No	Asymmetric; distal > proximal; axial	4/5	4+/5	NA	NA	5-/5	5-/5	4/5	4/5
**47**	Intermediate	43	Difficulty in jumping and running	Ambulant	63	Lower limb hypertrophy	Yes	No	Proximal lower limbs and axial	5/5	5/5	4-/5	4-/5	5/5	5/5	5/5	5/5

Table S3. Cardiac status in DMD heterozygous women.

ID	*Muscle weakness severity*	*EDV (ml/m²)*	*EF %*	*WMA*	*Strain %*	*Age at cardiomyopathy onset*	*Cardiomyopathy*	*ECG*	*Conduction/ Rhythm abnormalities*	*Heart failure symptoms*	*Cardiac therapy*
1	Asymptomatic	66	57	0	-16	-	No	Normal	No	No	No
2	Mild	41	61	0	-19	-	No	Normal	No	No	ACEi
3	Severe	50	65	NA	NA	-	No	Normal	No	No	No
4	Intermediate	41	56	0	NA	-	No	Normal	No	No	No
5	Severe	51	49	1	NA	NA	Yes	Left anterior fascicular block	Left anterior fascicular block	No	BB, ARB, diuretic
6	Asymptomatic	56	61	0	-20	-	No	Normal	Incomplete right bundle branch block	No	No
7	Severe	50	57	0	NA	-	No	Normal	No	No	ACEi
8	Asymptomatic	50	60	0	NA	-	No	Normal	No	No	No
9	Intermediate	51	64	0	-19	-	No	Normal	No	No	No
10	Intermediate	80	56	1	NA	74	Yes	Normal	Supraventricular and ventricular ectopic beats	No	ACEi, diuretic
11	Intermediate	54	52	1	-16	NA	Yes	Normal	No	No	ACEi
12	Asymptomatic	60	58	0	-20	-	No	Normal	No	No	No
13	Asymptomatic	57	56	0	-17.4	-	No	Normal	No	No	No
14	Asymptomatic	44	71	0	NA	-	No	Normal	No	No	No
15	Mild	41	61	0	NA	-	No	Normal	No	No	No
16	Asymptomatic	86	45	1	NA	32	Yes	Normal	Episode of VT	No	BB, ACEi, ASA
17	Asymptomatic	50	67	0	-20	-	No	Normal	No	No	No
18	Asymptomatic	57	53	0	-18	-	No	Normal	No	No	No
19	Asymptomatic	NA	68	NA	NA	-	No	NA	NA	No	No
20	Asymptomatic	49	64	0	-20	-	No	Normal	No	No	No
21	Mild	65	38	1	NA	40	Yes	Normal	Implantable cardiac defibrillator at 52 years of	Yes	BB, ACEi

									age due to sVT		
22	Asymptomatic	42	51	0	NA	-	No	Normal	No	No	No
23	Asymptomatic	52	65	0	-22	-	No	Normal	No	No	No
24	Asymptomatic	55	65	0	NA	-	No	Normal	No	No	No
25	Asymptomatic	61	60	0	-19	-	No	Normal	No	No	No
26	Asymptomatic	NA	NA	NA	NA	NA	NA	NA	NA	No	No
27	Severe	122	32	1	NA	62	Yes	Atrial fibrillation	Implantable cardiac defibrillator at 63 years of age; atrial fibrillation	Yes	Anti-arrhythmic, diuretic, anticoagulant
28	Mild	38	54	0	-15	-	No	Normal	No	No	No
29	Severe	70	43	1	NA	49	Yes	Normal	Supraventricular and ventricular ectopic beats	No	Beta blocker, ARB
30	Asymptomatic	59	54	0	-19	-	No	Normal	No	No	No
31	Mild	75	65	1	-19	48	Yes	Normal	Paroxysmal atrial fibrillation	No	Anticoagulant, anti-arrhythmic, ACEi, diuretic
32	Asymptomatic	50	62	NA	NA	-	No	NA	NA	No	No
33	Asymptomatic	33	78	0	NA	-	No	Normal	No	No	No
34	Mild	45	57	0	NA	-	No	Normal	No	No	No
35	Severe	58	60	0	NA	-	No	Normal	No	No	ARB, CCB
36	Severe	36	61	0	NA	-	No	Normal	No	No	ACEi, CCB
37	Asymptomatic	51	57	0	NA	-	No	Normal	No	No	No
38	Asymptomatic	37	57	0	NA	-	No	Normal	No	No	No
39	Mild	60	62	0	NA	-	No	Normal	No	No	No
40	Asymptomatic	66	61	0	NA	-	No	Normal	No	No	No
41	Asymptomatic	42	65	0	NA	-	No	Normal	No	No	No
42	Asymptomatic	NA	NA	NA	NA	NA	NA	NA	NA	No	No
43	Asymptomatic	NA	NA	NA	NA	NA	NA	NA	NA	No	No

44	Asymptomatic	42	70	0	NA	-	No	Normal	No	No	No
45	Intermediate	42	69	0	NA	.	No	Normal	No	No	No
46	Asymptomatic	60	60	NA	NA	-	No	Normal	Ventricular ectopic beats	No	No
47	Intermediate	49	45	1	NA	54	Yes	Normal	Supraventricular ectopic beats	No	BB, ACEi

ACEi: ACE inhibitors; AFB: anterior fascicular block; ARB: angiotensin receptor blockers; ASA: acetylsalicylic acid; BB: beta blockers; CCB: calcium channel blockers; ECG: electrocardiogram; NA: not available. sVT: sustained ventricular tachycardia. WMA: wall motion alterations (0 no, 1 yes).

Table S4. Dystrophin protein expression and XCI pattern in DMD heterozygous women; age at muscle biopsy (years) and age at blood collection for XCI assessment (years) are reported. NA: patients with unknow percentage of dystrophin negative fibers, dystrophin amount. Homozygote refers to the inability to establish XCI phase due to equal number of CAG repeats in the first exon of the AR gene.

ID	MUTATION(S)	PERCENT OF DYSTROPHIN NEGATIVE FIBRES	DYSTROPHIN AMOUNT (% OF NORMAL)	X-INACTIVATION	AGE AT MUSCLE BIOPSY	AGE AT BLOOD COLLECTION FOR XCI ASSESSMENT
**1**	del Dp427c-45	NA	NA	78	-	44
**2**	dup 3-43	NA	NA	79	-	57
**3**	del 51	NA	NA	77	-	57
**4**	del 44	NA	NA	Homozygote	-	-
**5**	dup 28-43	NA	NA	80	-	67
**6**	c.2548G>T	NA	NA	91	-	45
**7**	c.2302C>T	10	49	Homozygote	55	-
**8**	c.2656C>T	11	62	89	36	35
**9**	del 10-21	18	27	92	2	32
**10**	c.7222_7223del	5	50	63	69	74
**11**	c.2548G>T	NA	NA	61	-	66
**12**	del 8-43	NA	NA	52	-	32
**13**	c.2555-2556delG	NA	NA	90	-	44
**14**	c.2285A>C; c.4495C>T	NA	NA	75	-	34
**15**	c.8879G>A	NA	NA	54	-	45
**16**	c.6805C>T	NA	NA	52	-	45
**17**	del 52	NA	NA	71	-	39
**18**	c.1990C>T	NA	NA	74	-	40
**19**	del 49-52	NA	NA	99	-	40
**20**	del 8-9	NA	NA	61	-	50
**21**	dup 6-7	2	45	64	28	55
**22**	del 44-47	NA	NA	78	-	44
**23**	c.9685delT	NA	NA	61	-	51
**24**	dup 3-7	NA	39	Homozygote	3	-
**25**	c.3151C>T	NA	NA	81	-	44
**26**	del 61-63	NA	NA	73	-	49
**27**	del 49-54	10	91	100	38	55
**28**	del 49-50	NA	NA	62	-	64
**29**	del 49-50	NA	6	100	30	56
**30**	del 49-50	NA	NA	51	-	37
**31**	del 45-52	NA	NA	70	-	56
**32**	c.1150-1G>T	NA	NA	52	-	35
**33**	c.2168+1G>A	NA	NA	84	-	49

**34**	c.6591delA	NA	NA	51	-	20
**35**	c.1507G>T	14	40	95	36	53
**36**	del 46-55	NA	NA	56	-	59
**37**	del 10-11	8	50	Homozygote	8	-
**38**	15 Mb inversion in g Xp21.1	15	49	77	47	47
**39**	dup 51-67	NA	NA	54	-	22
**40**	del 49-54	NA	NA	51	-	46
**41**	c.7310-19A>G	NA	27	90	4	5
**42**	del Dp427m-47	NA	NA	Homozygote	-	-
**43**	c.1507G>T	12	68	62	4	21
**44**	c.358-2A>G	NA	NA	Homozygote	-	-
**45**	del 45-50	99	0	97	16	50
**46**	c.2950-2A>C	NA	NA	Homozygote	-	-
**47**	dup 51-67	98	8	88	43	59
